# WhatsApp messenger as a tool to supplement medical education for medical students on clinical attachment

**DOI:** 10.1186/s12909-017-0855-x

**Published:** 2017-01-06

**Authors:** Lewis Raiman, Richard Antbring, Asad Mahmood

**Affiliations:** Queen Mary University of London, Mile End Rd, London, E1 4NS UK

## Abstract

**Background:**

Instant messaging applications have the potential to improve and facilitate communication between hospital doctors and students, hence generating and improving learning opportunities. This study aims to demonstrate the feasibility and acceptability of instant messaging communication to supplement medical education for medical students whilst on clinical attachment.

**Methods:**

A total of 6 WhatsApp Messenger (WhatsApp Inc.) groups were created for medical students on clinical attachment. These were used to provide communication within Problem Based Learning (PBL) groups for a duration of 8 weeks. The frequency and type of communication were recorded. Students’ opinions were evaluated through a structured interview process at the end of the study period. A thematic analysis was performed on the content of the instant messaging groups and on the results of the structured interviews.

**Results:**

All of the participants were active in their respective messaging groups (19 students and 6 tutors). A total of 582 messages, 22 images and 19 webpage links were sent. Thematic analysis on content of the instant messaging groups identified the following themes: organisational, educational and social. Thematic analysis on the content of interviews identified themes such as the ease of use of instant messaging, benefit of instant messaging to foster understanding and learning, and the ability to access recorded discussions.

**Conclusion:**

The findings of this study illustrate a method by which communication within PBL groups can be facilitated by the use of instant messaging. The results indicate the feasibility and acceptability of WhatsApp Messenger in supplementing PBL teaching for medical students, and provides a framework for studies to investigate use amongst larger cohorts of students.

## Background

Smartphones are an important part of modern life enabling access to the internet through 4G, personalised directions via GPS, and even the ability to share pictures and videos on instant messaging applications. Smartphone ownership in the UK has increased dramatically with 76% of adults owning a smartphone, rising to 90% when considering the 16–24 year-old demographic [1]. It is therefore unsurprising that the vast majority of medical students own a smart phone and that instant messaging applications are becoming a more popular communication tool for students compared to emails [2, 3].

The use of mobile applications in medical and dental education has been shown to increase student participation, enhance the feedback process and improve communication between student and tutor [4–7]. Recently, there has been increased research into mobile Learning (mLearning), and specifically the use of instant messaging services. Using WhatsApp Messenger, an instant messaging application, in the primary health care education setting has demonstrated a number of benefits for undergraduate nurses. These include the usefulness of the application for integrating theory and clinical practice; increasing the availability of resources for test preparation and providing a platform for clarification of uncertain aspects of the course [8]. No study available to date has demonstrated the feasibility and acceptability of the use of instant messaging applications for medical students in the secondary care environment.

The hospital environment provides unique opportunities and challenges in the provision of medical education. Effective communication is necessary to take advantage of learning opportunities that are often sporadic. However, communication systems within the majority of UK hospitals are outdated, using old technology such as pager systems [9]. Almost all health professionals in the United Kingdom now carry and use smartphones, improving communication within the hospital environment [10]. Instant messaging applications have the potential to improve and facilitate communication between hospital doctors and students, hence generating and improving learning opportunities.

This study aims to demonstrate the feasibility and acceptability of instant messaging communication to supplement medical education for 3^rd^ year students whilst on clinical attachment through quantitative and qualitative analysis.

## Methods

All 3^rd^ year medical students attending medical and surgical clinical rotations and Problem Based Learning (PBL) facilitators at The Princess Alexandra Hospital, Harlow, UK from January to March 2016 and April to June 2016 participated in this study. A total of 19 students were on rotation during this time frame and provided informed consent to participate in the study.

Eligible students were allocated to one of 3 tutor groups dependent on the module they were studying. Each PBL session and each WhatsApp Messenger group was facilitated by 1 tutor. There were therefore 3 tutors for each study period.

The tutor held an introductory session with the students where the instant messaging groups were set up and the guidance from the General Medical Council (GMC) of the use of social media was explained to participants [11]. The groups met weekly for their usual PBL sessions, where the case was discussed amongst the students. These were run according to the medical school’s expectation of the structure and content of a PBL feedback session.

The study lasted for two 8-week periods, with a final feedback session at the end of each period where the students (*n* = 19) were interviewed with a structured approach to evaluate their experiences and thoughts using WhatsApp Messenger discussion group to supplement their learning experience.

### Analysis

Data was extracted from the WhatsApp Messenger groups and analysed in Microsoft Office Excel 2013. We looked at the frequency of communication, the degree of participation from group members and the content of the conversation. The content of the WhatsApp Messenger groups was analysed and grouped into separate themes.

## Results

### Quantitative results

**Fig. 1:**
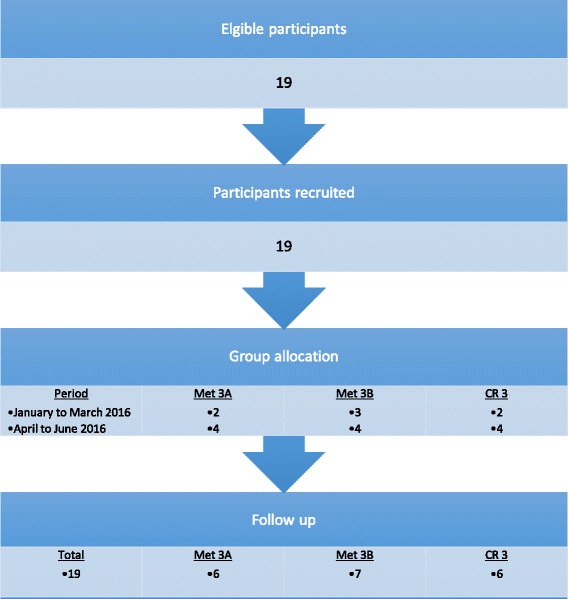
Flow diagram of the study methods. Met3A metabolism (gastroenterology and general surgery), CR3 (cardiology and respiratory), Met3B (endocrinology, nephrology, and urology)

Of the 19 participants enrolled, 0 were lost to follow-up providing 19 participants for interview and analysis (Fig. 1).

A total of 582 messages, 22 images and 19 webpage links were sent. Out of the 582 messages, 331 came from students, and 251 from tutors (Table 1).

**Table 1:** The amount of messages posted, based on subject

Group	Total Messages	Total Images	Total web page links	Total content
MET3A	267	9	2	278
CR3	135	13	4	152
Met3B	180	10	13	203
Average	194	11	6	211

**Fig. 2:**
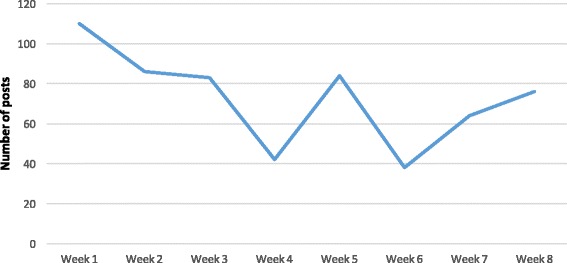
The number of posts per week

All students were active in initiation and participation of conversation within the groups. The range of posts per week varied between 38 and 110 (Fig. 2). GMC guidance was followed.

### Qualitative results

A thematic analysis was performed on the content of the WhatsApp groups, as well as the response from interviews. All the students involved in the study (*n* = 19) attended the final interview session.

#### Content themes

**Theme 1: Organisational**

A prominent theme that was identified was the use of WhatsApp as a means of organising PBL sessions. This was true for both tutors and students:

“*Oh ok thanks [Tutor name], yeah twelve is fine for us… We have that as a designated slot” (Student 1)*

The messenger service was commonly used to rearrange sessions. For students this was often due to scheduling problems:

*“Hi [Tutor name] we’re actually back in London for a week of lectures etc. so aren’t around for PBL but we’re back in Harlow for the 16* ^*th*^ *” (Student 2)*

*“Hey guys, would it be possible to do PBL slightly earlier today? [Surgeon name] asked us to go into surgery this afternoon!” (Student 3)*

And for tutors due to managing workload:

*“I’m flying back tomorrow. If you guys want could do the session at some point on [Thursday]?” (Tutor 1)*

**Theme 2: Educational**

There were a variety of educational interactions; including tutors asking questions:

*“What is involuntary guarding and why is it an important sign?” (Tutor 2)*

*“Is involuntary guarding where the patient tenses up there [sic] abdominal muscles on palpation? A sign of acute abdomen?” (Student 4)*

Furthermore, students often asked questions to clarify concepts:

*“Cross match and Group & Save not sure exactly when you’d do one or the other or both?” (Student 4)*

*“Yeah I agree with you [Student 1]… No idea why…” (Student 5)*

*“Crossmatch is when the blood that is prepared for the patient is checked against the blood group of the patient. The blood group is obtained by a group and save” (Tutor 2)*

**Fig. 3:**
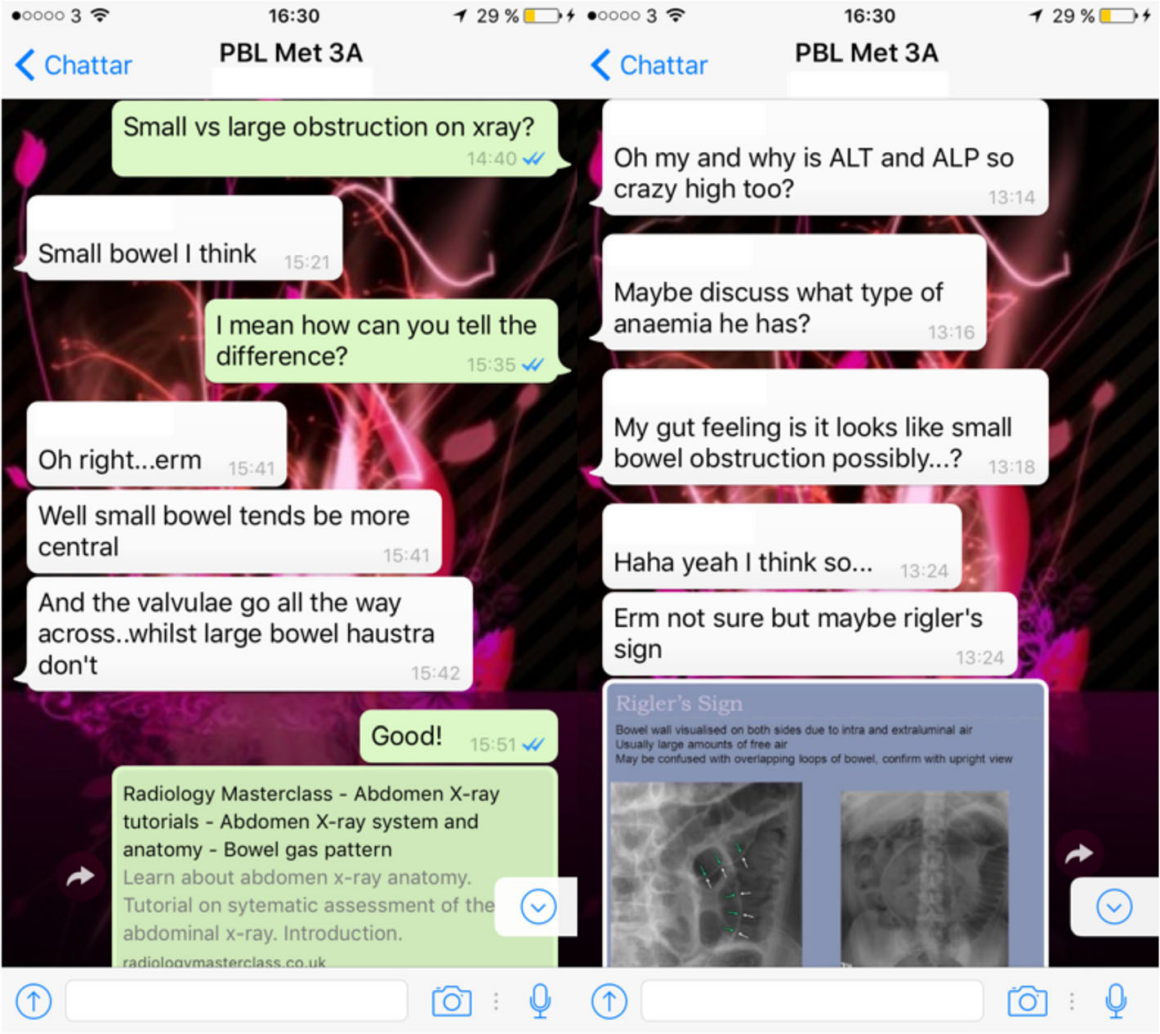
Examples of discussions and exchange of resources between students and tutor (names of participants have been removed)

Discussion amongst students and tutors frequently occurred and often involved sharing of resources (Fig. 3):

Interestingly, the WhatsApp groups were often used to communicate sporadic learning opportunities:

*“There’s an ABPI that needs doing on [Ward name] if you want to join” (Tutor 2)*

*“Oooooh ok. Yeah, I’ll head down in 5!” (Student 6)*

Students also sometimes asked for further learning opportunities outside of the PBL setting:

*“[Tutor name], are there any patients I can clerk in A&E?” (Student 7)*

**Theme 3: Social**

**Fig. 4:**
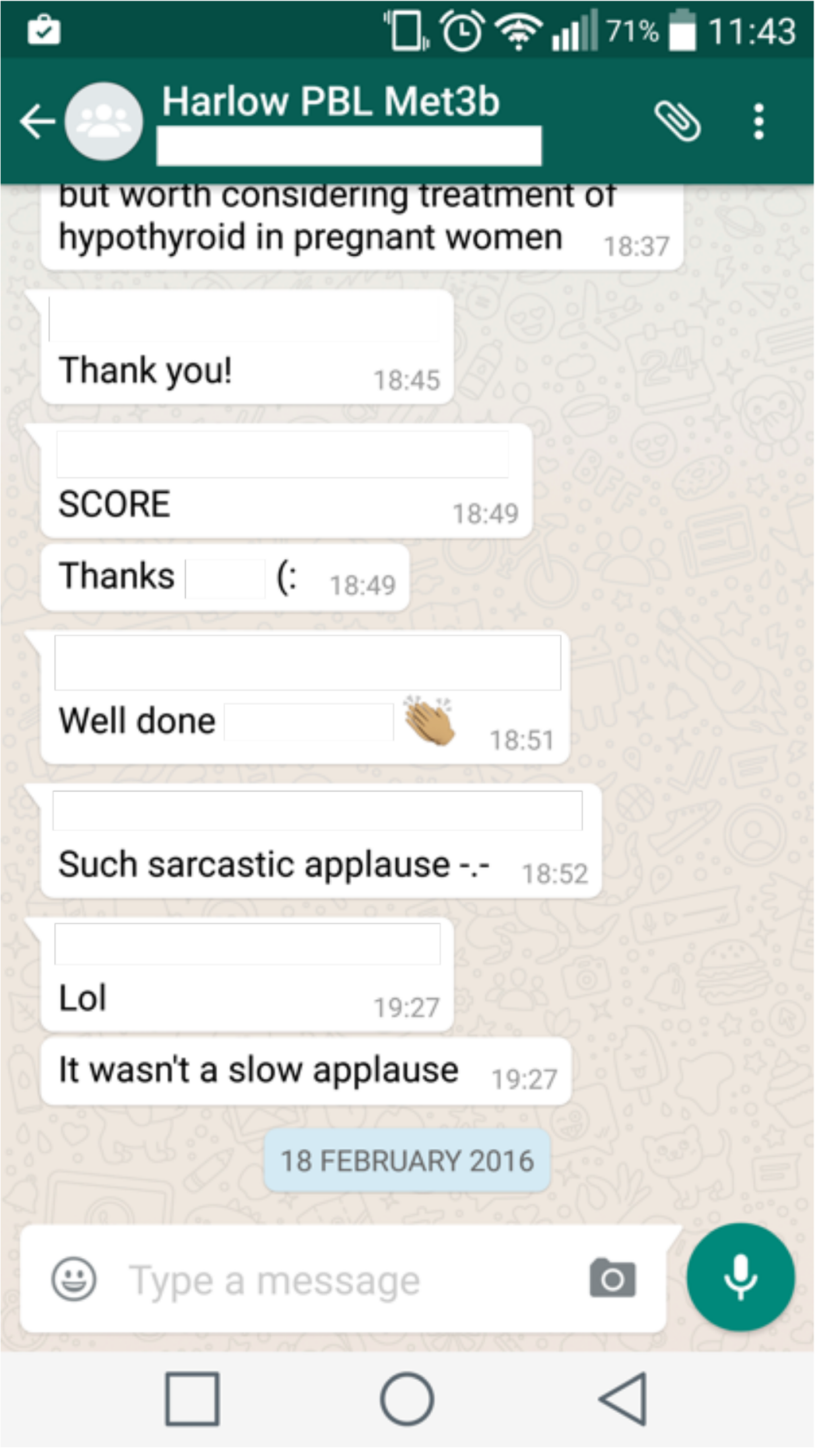
Examples of social interaction between students (names of participants have been removed)

A social presence was ubiquitous in the groups and often involved informal language and the use of emoticons (Fig. 4):

A positive social atmosphere was fostered and was seen throughout the groups:

*“Hope the [Internal Continuous Assessment] went well. Enjoy a well deserved weekend!!!” (Tutor)*

*“Thank you!” (Student 7)*

*“Thanks [Tutor name], you too!” (Student 8)*

*“Thank you [Tutor name]!” (Student 9)*

#### Interview themes

Following each 8-week period, a feedback session was conducted with all the students to evaluate their thoughts and experiences about the use of WhatsApp Messenger to supplement PBL learning. The content of the responses to these interviews was subsequently grouped into themes.

**Theme 1: The ease of use of instant messaging**

All of the participants reported feeling familiar with the use of WhatsApp Messenger and found it easy to use. None of the participants required introduction or tutorials to use the messaging platform, instead using it with confidence, due to using the application in their everyday life:

*“I felt really confident in using instant messaging in PBL and I feel as though it benefitted my learning” (Student 4)*

**Theme 2: Benefit of instant messaging to foster understanding and learning**

The value of WhatsApp Messenger in supplementing the PBL process formed the majority of interview content. Responses were largely focused on improved communication and understanding of the subject matter. A number of students reported being more participant in the sessions as a result of instant messaging:

*“I thoroughly enjoyed the use of instant messaging to continually develop the learning objectives and communicate within the PBL group” (Student 5)*

*“I found the instant messaging component of the PBL really useful. It allowed more time to spend discussing.” (Student 4)*

*“Messaging made the session far more stimulating. We were able to focus on specific problems we had in the session. This made the whole learning experience far more useful and specific to us” (Student 10)*

**Theme 3: Sharing resources electronically**

Resources were frequently shared within the groups, something that was mentioned as being beneficial during the interviews. Tutors expressed the value of using media to demonstrate and explain difficult concepts. Being able to direct students towards reliable and credible sources, such as National Institute of Clinical Health and Excellence (NICE) guidance was also mentioned.

*“Sharing resources* via *messaging and ad hoc explanations throughout the week were highly appreciated.” (Student 5)*

**Theme 4: Accessing recorded discussions**

Students highlighted the practicality of being able to access previous conversations and resources and being able to refer back to them. Furthermore, the value of being able to access this at all times was expressed.

*“It is very helpful to look back at PBL and related links are easy to access. Work is split evenly between the group.” (Student 11)*

**Theme 5: Generating learning opportunities outside PBL**

Interviews revealed students’ appreciation of being able to contact tutors about further learning opportunities. This often occurred on an ad-hoc basis, and led to an increase in clinical exposure for students. Tutors, likewise, praised the ability of quickly being able to contact students for impromptu learning opportunities:

*“It enabled us to have more ward based teaching. This was a highly beneficial bonus.” (Student 7)*

**Theme 6: Intrusiveness of instant messaging**

None of the students felt the use of instant messaging was intrusive. One student expressed the benefit of being of able to control notifications on their mobile phone, and only allowing notifications during study hours. Several students and tutors viewed the messages from the group as comparable to the social messages they received from their other contacts in the application, and viewed the interactions as positive:

*“I did not find instant messaging intrusive to everyday life. The instant messaging component allowed to being able to respond to messages when I found it convenient” (Tutor 3)*

**Theme 7: Lack of face-to-face interaction.**

One tutor stated that he found the transition towards teaching over instant messaging as difficult, due to the absence of non-verbal communication. He therefore highlighted a concern about the difficulty to identify students who were not adequately understanding concepts.

*“One of the negative aspects of the WhatsApp messaging groups was that I was unable to see the students when was interacting with them. The lack of body language left me uncertain sometimes of whether the student understood my explanations.” (Tutor 2)*

## Discussion

This study explored the use of WhatsApp Messenger as a tool to facilitate communication between students and doctors and its value in supplementing medical education for medical students on clinical attachment. To our knowledge, this is the first time a study has investigated the use of WhatsApp Messenger as a communication tool between students and tutors in this context. The results of this study demonstrate how instant messaging groups can be a useful tool for students in the PBL process through promoting of media sharing, improving communication, generating learning opportunities and providing a record of discussions.

The established popularity of WhatsApp Messenger as a social communication tool aided its successful integration into teaching. This was the main reason why it was chosen as the application of choice in this study. The familiarity of the use of the application meant that no training had to be organised. Furthermore, there was no additional financial cost incurred by the use of WhatsApp Messenger, due to the fact that students and tutors already had the application installed on their phones. Whilst the application previously incurred a yearly charge of 69 pence, it was made completely free during this study by WhatsApp Inc. Other messaging platforms exist, some being free at the point of use, such as Messages (Apple Inc.) or Facebook Messenger (Facebook Inc.). These applications have comparable functionalities to WhatsApp Messenger, but do have some disadvantages, such as lack of cross-platform connectivity and the need to have a Facebook account respectively. Facebook Messenger does however have the added benefit of the user being able to access and create content on a desktop computer. Unlike these applications, WhatsApp Messenger, has recently added an end-to-end encryption system, providing a secure platform for communication.

Students remained engaged throughout the study period. The first week was the most active study period, with 110 messages being posted, reflecting the establishment of group dynamics. In subsequent weeks the number of posts stabilised, averaging about 60 posts per week. Engagement is illustrated by the fact that the majority of posts (331 out of 582) within the study period were produced by students rather than tutors. This also highlights the fact that students felt comfortable asking questions to each other and tutors, reflecting the fact that the instant messaging platform may serve to flatten hierarchies. Studies into the use of WhatsApp in the healthcare environment has demonstrated the effectiveness in its use as a communication tool, by overcoming human factor barriers to effective communication. It also allows for increased connectedness between team members [10]. These results echo the thoughts expressed by students in the feedback interviews.

Students found that WhatsApp Messenger provided a platform to share results and links to resources whilst studying, fostering a collaborative approach to learning. The ability of being able to quickly find and utilise resources, whilst simultaneously participating in an interactive group discussion on WhatsApp Messenger, posting images, documents and web page links that students find useful and wish to share is unique to the smartphone environment. Being able to access the classroom environment when convenient for students also allows for more opportunities for discussion of learning material and follows the enquiry orientated method that is central to PBL [12]. This is demonstrated in the WhatsApp Messenger groups where students were able to ask questions and receive information at any point during the week between sessions. This was highlighted as one of the advantages in the interviews, along with the ability to refer back to this information at any time. Similar benefits were observed in a study into the benefits of WhatsApp Messenger for nursing students in primary health care education, [12] and other studies involving e-learning [13]. The record of communication also allows for tutors to reflect back on student participation rates and the effectiveness of facilitating discussions. Furthermore, the group discussions were not solely limited to PBL topics. Impromptu bed side teaching, practical procedure teaching and shadowing was arranged within the groups. Our results demonstrated that the WhatsApp messaging platform enabled students to take advantage of sporadic learning opportunities within the hospital setting, that otherwise may have been missed. Similar effects in the traditional classroom have been seen through the introduction of more online learning [14]. Furthermore, the use of instant messaging between students and tutors provided an effective and rapid method of arranging and rearranging sessions, compared to other methods such as e-mail.

WhatsApp Messenger sends push-notifications to the user when a new message is received. This may provide a distraction for the user whilst at work, and may be experienced as intrusive due to being connected all the time. Whilst there is a lack of studies into this issue in the context of mobile learning, the problem has been raised in studies in blended learning, where extending the classroom to the home has been seen as invasive [15]. We did not observe any of these concerns during our study. One student mentioned the ability of controlling these notifications, and therefore accessing the messages at a convenient time. Some tutors might also not want to provide their mobile phone numbers to students or vice-versa, an issue not seen in this study.

The absence of face-to-face interaction between tutor and student during discussion of learning objectives and difficult concepts may be seen as a potential drawback by using WhatsApp Messenger for this process, as expressed by one tutor. Previous studies in blended learning have expressed concern that some students may be more likely to avoid the educational material if it is presented outside the classroom [16]. In small groups as in our study, it was clear to tutors whether students were engaged in the groups, as each chat entry is tagged with a student name. This is a clear benefit to the online content of blended learning, where it is often hard to know whether students are engaging in the content [16]. This study involved a combination of instant messaging and face-to-face teaching through feedback sessions. The students involved in our study reported that they felt confident in asking questions and raising concerns when they did not fully understand concepts. Whilst this may not be true for all students, the face-to-face teaching provided by feedback sessions allow tutors to confidently identify any issues the students may have understanding the content. This is vital, as there are some concerns that active engagement in learning of online material may be downgraded versus learning in the traditional format [13].

Hospital trusts in the UK have traditionally opposed using instant messaging as a means for clinicians to discuss patient details due to potential confidentiality issues. However, with the presence of end-to-end encryption, as well as password or finger print protected smartphones, there remains few arguments against being able to use a platform such as WhatsApp to refer and discuss patients. This would further enhance connectivity between clinicians, and aid communication within and between teams. The added benefits of the instant messaging platform such as a record of discussion, and the ability to share media such as radiological imaging may also provide useful if a clinician does not have access to a computer at the time of communication. The use of WhatsApp as a tool to discuss patients in the clinical environment has previously been studied [10], but needs to be further investigated in terms of its applicability to the hospital environment as a whole.

### Limitations

The small group sizes (*n* = 3-5) and overall sample size (*n* = 25) of the study may be seen as a limitation to the results. However, we believe that small groups promoted a positive group dynamic, which increased student engagement and participation in the WhatsApp Messenger groups. The impact of larger group sizes on the degree of engagement and participation of students remains unclear. A larger overall sample size would improve the applicability and ability to generalise these results to other medical students. Future efforts towards applying these methods onto larger and more diverse cohorts should aim to encourage and maintain student engagement.

Whilst beyond the scope of the study, testing the students’ understanding and retention of the content taught via WhatsApp versus traditional face-to-face teaching would have been useful. This could give insight into whether there are any differences in the degree of understanding students achieve between the traditional PBL approach and our, novel, approach. A cross-over design, where the students would have 8 weeks of traditional PBL teaching followed by 8 weeks of teaching including WhatsApp would have also highlighted the differences to students more vibrantly, and may have led to greater interview content on the positives and negatives of our novel teaching method.

## Conclusion

The findings of this study illustrate a method by which communication and education within PBL groups can be facilitated by the use of instant messaging. The results indicate the utility, feasibility and acceptability of WhatsApp Messenger in supplementing PBL teaching for third year medical students, and provides a framework for studies to investigate its use amongst larger cohorts of students. The study also highlights the potential use of WhatsApp as a tool to enhance communication between clinicians, and its applicability in the clinical environment needs to further investigated.
